# Assessment of the nano-mechanical properties of healthy and atherosclerotic coronary arteries by atomic force microscopy

**DOI:** 10.1098/rsif.2023.0674

**Published:** 2024-02-07

**Authors:** Fotios Savvopoulos, Michael C. Keeling, Daniele Carassiti, Nicholas A. Fogell, Miten B. Patel, Jarka Naser, Núria Gavara, Ranil de Silva, Rob Krams

**Affiliations:** ^1^ Department of Bioengineering, Imperial College London, London SW3 6LR, UK; ^2^ National Heart and Lung Institute, Department of Medicine, Imperial College London, London SW3 6LR, UK; ^3^ School of Engineering and Materials Science, Queen Mary University of London, London E1 4NS, UK; ^4^ Unit of Biophysics and Bioengineering, Medical School, University of Barcelona, Barcelona 08007, Spain

**Keywords:** atomic force microscopy, coronary arteries, atherosclerosis, nano-indentation, collagen microstructure, mechanical properties

## Abstract

Nano-indentation techniques might be better equipped to assess the heterogeneous material properties of plaques than macroscopic methods but there are no bespoke protocols for this kind of material testing for coronary arteries. Therefore, we developed a measurement protocol to extract mechanical properties from healthy and atherosclerotic coronary artery tissue sections. Young's modulus was derived from force-indentation data. Metrics of collagen fibre density were extracted from the same tissue, and the local material properties were co-registered to the local collagen microstructure with a robust framework. The locations of the indentation were retrospectively classified by histological category (healthy, plaque, lipid-rich, fibrous cap) according to Picrosirius Red stain and adjacent Hematoxylin & Eosin and Oil-Red-O stains. Plaque tissue was softer (*p* < 0.001) than the healthy coronary wall. Areas rich in collagen within the plaque (fibrous cap) were significantly (*p* < 0.001) stiffer than areas poor in collagen/lipid-rich, but less than half as stiff as the healthy coronary media. Young's moduli correlated (Pearson's *ρ* = 0.53, *p* < 0.05) with collagen content. Atomic force microscopy (AFM) is capable of detecting tissue stiffness changes related to collagen density in healthy and diseased cardiovascular tissue. Mechanical characterization of atherosclerotic plaques with nano-indentation techniques could refine constitutive models for computational modelling.

## 1. Introduction

Coronary plaque rupture is the primary mechanism for coronary thrombosis in patients with acute coronary syndrome (ACS) [1]. According to retrospective histopathological reports [2–5], rupture-prone advanced plaques tend to possess the following characteristics: high plaque burden, expansive remodelling, a lipid-rich necrotic core, a collagen-rich fibrous cap infiltrated with immune cells, calcifications and intraplaque haemorrhage. *In vivo* morphological descriptors of rupture-prone plaques such as stenosis severity or plaque burden based on angiography and intracoronary imaging alone have limited clinical predictive value of a future major adverse cardiac event (MACE) [1].

Experimental and clinical data show that biomechanical forces have an important role in the natural history of coronary atherosclerosis [6–8]. Computational approaches for the analysis of the biomechanics of coronary plaques have evolved over the last 15 years. Coupled approaches, such as fluid–structure interaction (FSI) modelling, offer the most physiologically relevant framework to analyse coronary plaque biomechanics, given the structural complexity and interplay of endothelial shear and strain mechanobiology [9].

Reliability of tissue mechanical models are dependent on structural segmentation and material properties of the diseased coronary wall. *In vivo* methods for deriving material properties with ultrasound elastography [10] and magnetic resonance elastography [11] would be desirable, but they lack large-scale validation and sufficient resolution to capture plaque heterogeneity. *Ex vivo* methods for extracting material properties dominate the landscape of material testing for atherosclerotic plaques. Although such methods can capture the most salient feature of arterial response, tissue hyperelasticity, they have several limitations. First, histopathological studies have demonstrated the marked heterogeneity of plaque tissue with soft (lipid) and stiff (collagen, calcium) components in ruptured coronary plaques being situated a few microns apart [3,4]. Even on that scale, collagen displays an anisotropic arrangement in the form of non-uniform fibre density and orientation. As a corollary, plaques possess heterogeneous plaque strength, and tensile methods are agnostic to local variations in plaque stiffness. Moreover, macro-mechanical testing may be partial to samples that are rigid enough to withstand arterial clamping.

Atomic force microscopy (AFM) is an alternative, *ex vivo*, material testing technique to derive local material properties by indentation [12,13]. By working on tissue sections, it is possible to study the relationship between histologically defined tissue composition and structure on local plaque mechanics. In contrast to tensile testing [14] there are no standardized protocols for this approach to material testing for coronary arteries. Evaluating experimentally induced human-like atherosclerosis in preclinical models would be an appropriate approach to develop and refine novel material testing protocols, the outputs of which could be incorporated into *in vivo* biomechanical models of healthy and diseased coronary arteries.

In this study, it is hypothesized that local mechanical properties of coronary arteries are dependent on local composition and structure. The aims of this study are to: (i) present a reproducible nano-indentation protocol for healthy and atherosclerotic coronary arteries, (ii) provide a robust co-registration framework with local collagen microstructure, and (iii) relate nano-mechanical properties to the underlying composition and structure of healthy and atherosclerotic coronary arteries.

## 2. Material and methods

### 2.1. Animal model, tissue cryopreservation and cryosectioning

Healthy (left anterior descending) and atherosclerotic (right coronary) arteries were sourced from a porcine model of atherosclerosis in hypercholesterolaemic *D374Y*-PCSK9 Yucatan minipigs [6,15]. The arteries were carefully excised from the heart muscle, were cut into 2 mm segments, embedded in OCT (Fisher Scientific, catalogue number: 23-730-571) and snap-frozen in isopentane (2-methylbunane, 99+%, Fisher Scientific, catalogue number: 10121820). Tissue blocks were cryo-sectioned at 20 µm thickness and picked up with gridded coverslips (Ibidi, Germany, catalogue no.: 10817). The coverslips were then placed into plastic Petri dishes (Merck, catalogue no: CLS430165) that were sealed tightly with parafilm (Merck, catalogue no: P7543) to avoid development of condensation. The samples were stored at −80°C until further use. Cryopreservation is known not to alter the mechanical properties of arterial tissue to a significant degree [16,17]. A total of seven animals were studied, four (12 vessels) pigs were not fed a high-cholesterol diet and three (nine vessels) pigs were fed a high-cholesterol diet.

### 2.2. Indentation testing

Nano-indentation measurements were conducted using a JPK Nano-Wizard 4 (JPK Instruments, Germany). The tissue samples were indented with a MNSL-10 (type F) probe (Bruker, Germany) with a *k* = 0.6 N m^−1^ nominal spring constant. The probes were calibrated with the thermal noise method [18]. Prior to measurement, the frozen tissue sections were rehydrated with physiological phosphate buffered saline (PBS). All measurements were conducted in liquid conditions at a controlled temperature (23°C). Indentations were applied in the longitudinal direction of the native vessel. The force threshold was chosen to ensure the indentation depth did not exceed 10% of the tissue thickness (linear regime). Viscoelasticity effects of arterial tissue were minimized by applying constant ramping of the indentation speed throughout the experiments.

**Figure 1.**
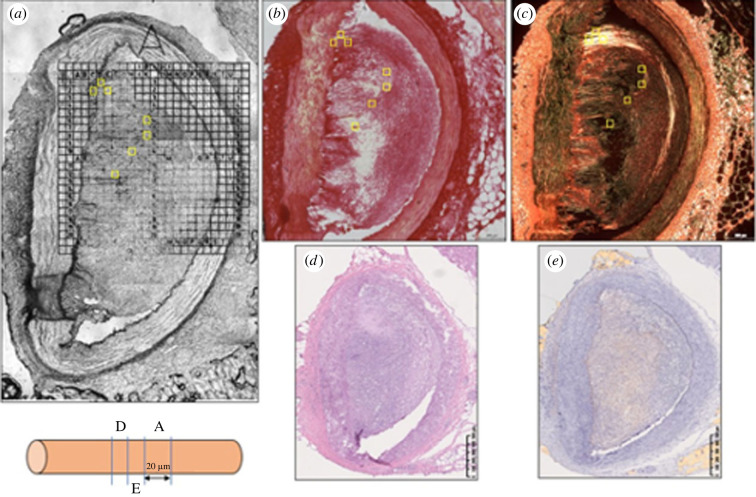
The process of co-registration of nano-indentation measurements in atherosclerotic coronary arteries with local collagen microstructure. (*a*) Brightfield image of a tissue section with indentation locations/ROIs (50 × 50 µm) shown in yellow squares, (*b*) The same section stained with Picrosirius Red and imaged with brightfield light with focus on quadrant A, (*c*) Same section stained with Picrosirius Red imaged with linearly polarized light with focus on quadrant A, (*d*) Adjacent section (10 µm) stained with Hematoxylin and Eosin, (*e*) Adjacent section (10 µm) stained with Oil-Red-O for lipid content.

Measurements were performed over multiple square (50 × 50 µm) regions of interest (ROIs). Within each ROI, the probe executed force indentations in a grid-like fashion with 10 µm step size (x-y distance between probed locations), resulting in 25 indentations (force curves). In healthy arteries, the intima-media layers were primarily targeted due to their biomechanical importance under physiological loading conditions [19]. Multiple locations within those layers were selected in an attempt to gather indentation data from locations with various collagen content. Slender, dark bands were targeted using the optical microscope arm of the AFM, which retrospectively were confirmed to be areas rich in collagen (electronic supplementary material, figure S1; figure 1). Within diseased tissue sections, areas in the Picroserius Red stained images devoid of collagen were considered lipid rich, and dense collagen area were considered cap area (figure 1*b,c*). It is believed that the presence of stiff (collagen, calcium) and soft (lipid) components in advanced plaques are source of stress concentrations that are precursors of plaque rupture [8].

Signal-to-noise ratio often dropped during the experiment due to probe contamination with extracellular matrix residue. Therefore, collagenase powder (type I from Clostridium Histolyticum, Sigma Aldrich, catalogue number: 9001-12-1) buffered in Hank's Balanced Salt solution (Sigma Aldrich, catalogue number: H2387) at a concentration of 3 mg ml^−1^ was used to decontaminate the probes at 37°C for 45 min.

### 2.3. Derivation of Young's modulus from force curves

The raw force curves were imported to JPKSPM Data Processing (v. 6.1) software. For a series of raw ‘force curves’ see electronic supplementary material, figure S2. A custom-made subroutine that was created from a list of standard curve processing operations was applied uniformly in the entire cohort of force curves. A Hertzian contact mechanics model was fitted to the experimental force curves in order to extract Young's modulus (E). Assuming no adhesion and friction forces, when a sample is probed with a pyramidal tip the force increases parabolically with respect to the indentation depth [20,21],

$$F=\frac{2}{\pi }\frac{E}{1-\nu^{2}}\tan(a)\delta^{2},$$

where *F*: force, E: Young's modulus, *ν*: Poisson's ratio, *α*: half opening angle of tip, *δ*: indentation depth. The root mean square (RMS) of the force residuals was used to assess the goodness of fit.

**Figure 2.**
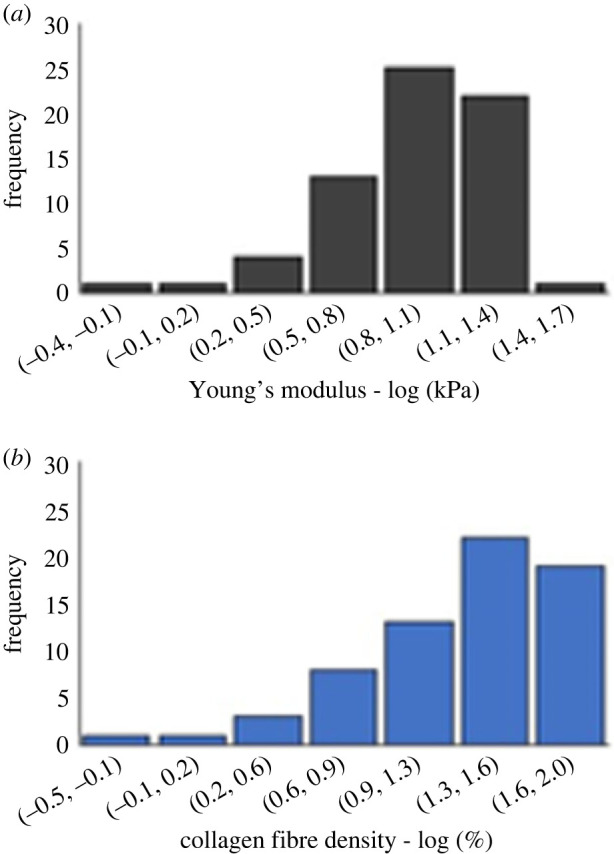
Frequency distribution of (*a*) Young's modulus and (*b*) collagen fibre density results for healthy coronary arteries. Frequency number corresponds to *n* = 67 indentation locations/ROIs in *n* = 14 healthy tissue sections. A representative value of stiffness was assigned to each indentation location by calculating the median Young's modulus from 25 force curves (within each ROI). This value was paired with one value of collagen fibre density (*n* = 67). Young's modulus was found to follow a lognormal distribution (Young's modulus, *p* = 0.045), whereas collagen fibre density did not (*p* = 0.2754).

### 2.4. Collagen staining, image acquisition, digitization and extraction of collagen metrics

After mechanical testing, the tissue was stained with Picrosirius Red according to routine histological protocols. Adjacent sections (cut at 10 µm thickness) were stained with Hematoxylin and Eosin (general pathology) and Oil-Red-O (lipids). The sections were analysed using an inverted light microscope Zeiss AxioObserver (Jena, Germany) equipped with a built-in automated acquisition system (TissueFAX i Plus, TissueGnostics GmbH, Austria). Acquisition was done with a 20× microscope objective (Zeiss EC Plan-Neofluar 20x/0.5). The microscope had built-in capacity for linearly polarized light imaging. Before each acquisition the polarizers were ‘crossed’ to achieve a black background. Image acquisition took place under identical conditions. The images were stored in a high-resolution format (.tiff) and were imported in ImageJ v. 1.53c (NIH, USA). A measure of the total (all types) collagen density was extracted by converting the polarized light image to greyscale. Greyscale images were subjected to an identical threshold level (level: 75/255) that resulted in binary images from which collagen fibre density was derived as the percentage of white pixels within a ROI. The threshold level was selected by validating the collagen content of the medial layer against measurements of collagen content from porcine coronary arteries derived with second harmonic generation microscopy [22] (electronic supplementary material). As the grid was not always visible in the dark-field image under polarized light (figure 1*c*), the brightfield, stained image—where the grid was visible (figure 1*b*)—was used as an intermediate image between the former and the AFM brightfield image (figure 1*a*) to enable the accurate co-registration of mechanical properties and collagen fibre density.

### 2.5. Statistical analysis

Each ROI consisted of 25 force curves. A Young's modulus value was extracted from each force curve. The median of the 25 values was assigned as a representative stiffness value of the indentation location/ROI. For the purposes of the co-registration, the median Young's modulus of each indentation location was paired with one value of collagen fibre density corresponding to the entire 50 by 50 µm area. The log-normalized Young's modulus and collagen fibre density frequency distributions were tested for normality using the Kolmogorov–Smirnov test.

The log-normalized pairs of Young's modulus and collagen fibre density were categorized into bins according to the following procedure. First, the collagen fibre density data were sorted from lowest to highest rearranging the Young's modulus values. A bin size of six data points was defined. Then for every six values of the collagen fibre density or Young's modulus data, the median value was calculated and replaced the former six values creating new pairs.

Comparisons between groups were done with non-parametric Wilcoxon ranked sum (for unpaired comparisons) and Wilcoxon signed rank (for paired comparisons) tests at 5% significance level. Statistical analyses were done in Matlab 2020b (Natick, USA).

## 3. Results

In total, 30 (*n* = 30) tissue sections were analysed. Fourteen (*n* = 14 sections, 1379 force curves in 67 ROI's) sections corresponded to healthy tissue, whereas 16 (*n* = 16, 1898 force curves) sections corresponded to atherosclerotic tissue.

**Figure 3.**
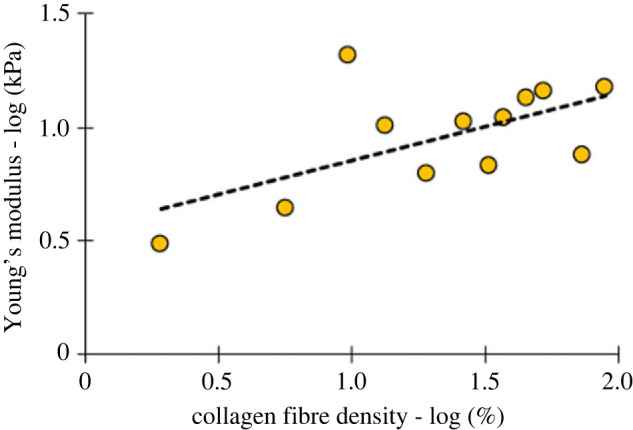
Scatter plot between log-transformed Young's modulus and collagen fibre density for healthy group (14 tissue sections, *n* = 67 co-registered data points). The co-registered datapoints were binned to 12 groups of six data points each. A significant positive correlation (Pearson's *ρ* = 0.61, *p* = 0.037) was found to relate the variables.

Within the healthy group, *n* = 67 regions of interest (50 × 50 µm areas consisting of 25 force curves each) resulted in *n* = 67 Young's modulus values, each of which were in turn co-registered with a single value of collagen fibre density, resulting in *n* = 67 co-registered data pairs. The frequency distributions of these variables were plotted individually. Young's modulus was found to follow a skewed lognormal distribution (*p* = 0.045) (figure 2*a*) whereas collagen fibre density did not (*p* = 0.2754) (figure 2*b*). When plotted against each other (figure 3), a significant positive correlation (Pearson's *ρ* = 0.61, *p* = 0.037) was found to relate the two variables.

**Figure 4.**
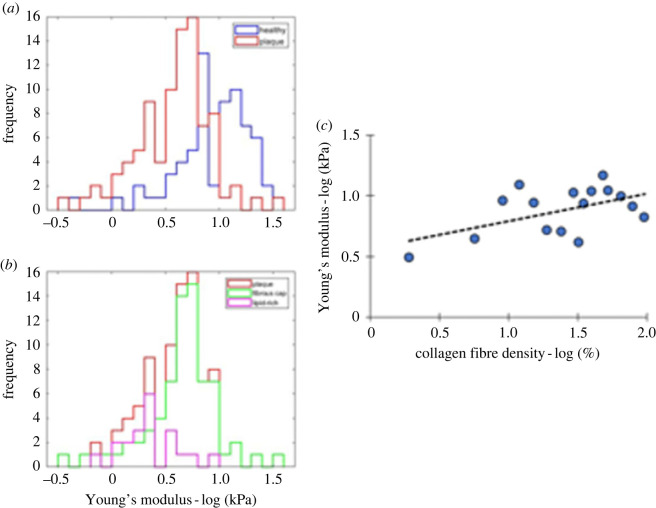
(*a,b*) Comparison of frequency distribution of Young's modulus among different histology groups; (*a*), (blue) healthy tissue; (*a*,*b*), (red) plaque; (*b*), (green) fibrous cap; (*b*), (magenta) lipid-rich necrotic core. (*c*) Scatter plot of co-registered collagen fibre density and Young's modulus measurements located in healthy coronary wall (*n* = 67) and fibrous cap (*n* = 24). The co-registered datapoints were binned to 17 groups of six data points each. A positive correlation (Pearson's *ρ* = 0.53, *p* = 0.036) was found to relate the variables.

Figure 4 shows a left shift in the distribution of Young's modulus (figure 4*a*) in atherosclerotic plaques compared with healthy arteries due to reduced values in both the lipid components and fibrous cap of plaques (figure 4*b*). In figure 4*c*, co-registered measurements from the region of the fibrous cap (*n* = 24) within diseased tissue sections were added to the dataset of the healthy group (figure 3) which showed a trend towards a positive correlation between Young's modulus and collagen fibre density (Pearson's *ρ* = 0.53, *p* = 0.036).

The mechanical properties of the healthy vessel wall (median = 11.0 kPa) were found to be significantly stiffer (*p* < 0.001) than plaque tissue (median = 4.4 kPa) (table 1). Within plaques, lipid-rich areas (median = 2.2 kPa, *n* = 392 force curves) were found significantly softer (*p* < 0.001) than areas rich in collagen such as the fibrous cap (median = 4.9 kPa, *n* = 1506 force curves). No statistical difference (*p* = 0.89) was found between measurements in the middle of the fibrous cap (median = 4.8 kPa, *n* = 868 force curves) and the cap shoulder (median = 5.1 kPa, *n* = 638 force curves). The stiffness of the fibrous cap (median = 4.9 kPa) was found to be almost half as much as that of healthy tissue (median = 11.0 kPa). The median collagen content of the fibrous cap was estimated as 16.2% (*n* = 24 locations), half that of healthy coronary media (32.4%, *n* = 67 locations).

**Table 1.** Summary of nano-mechanical properties (A) and collagen microstructure measurements (B) per histological group. Plaque group consists of measurements in lipid-rich necrotic core and measurements in the fibrous cap. Fibrous cap group consists of measurements in the fibrous cap shoulder and mid-cap. Discard rate is considered in the final number of processable force curves. Estimation of collagen content is shown in B. Due to technical difficulties with the staining procedure, collagen metrics for the diseased group were gathered from a limited number of tissue sections, thus histological classification of AFM measurements (A) was done on the basis of adjacent Hematoxylin and Eosin and Oil-Red-O sections. IQR: interquartile range.

		Α. nano-mechanical properties			Β. collagen microstructure	
		number of force curves	Young's modulus (kPa)		collagen density (%)	number of ROIs
			median	IQR		
healthy wall		1379	11.0	8.6	32.4	67
plaque		1898	4.4	2.8	—	31
lipid-rich necrotic core		392	2.2	1.7	2.7	7
fibrous cap		1506	4.9	2.9	16.2	24
	shoulder	638	4.8	3.4	17.8	14
	mid-cap	868	5.1	2.8	14.7	10

## 4. Discussion

Tensile (uniaxial/biaxial) [14], unconfined compression [23] or inflation testing have been extensively used to characterize the mechanical properties of advanced atherosclerotic plaques. The major limitations of macroscopic tensile methods is that they are agnostic to microscale variations in tissue stiffness and restricted to arteries of sufficient size. Material testing by indentation, such as atomic force microscopy, may be more suited to quantify local variations in plaque stiffness. Although it is a well-established technique to study cellular and bio-tissue mechanics [24,25], it is still in early stages of development for application to the study of arterial biomechanics. This high-resolution material testing method could aid the refinement of biomechanical constitutive models which are currently based on macroscopic testing.

In this work we developed a nano-indentation protocol for mechanical characterization of healthy and atherosclerotic porcine coronary arteries. We co-registered those properties to the local collagen microstructure with a robust framework utilizing coverslips with an imprinted 50 µm grid and we attained a total co-registration error of 4.02% (electronic supplementary material, §S1). We then used this framework to link the mechanical properties of healthy and atherosclerotic coronary arteries to the local collagen microstructure. To summarize our findings, plaque tissue was found to be softer than the healthy coronary wall. Fibrous cap areas were significantly stiffer than areas rich in lipid. Stiffness was independent of the location within the fibrous cap. The stiffness of the fibrous cap was half as that of the healthy coronary media. Young's modulus correlated positively with collagen content.

Our stiffness results for healthy coronary wall lie within the range reported in the literature. It is noteworthy that most studies have been conducted in elastic arteries (e.g. aorta [26–28], carotid artery [29]) which possess a different microstructure to muscular arteries (e.g. coronary), therefore the stiffness range is shifted towards higher values. Elastic arteries consist of many concentric elastic lamellae that are rich in elastin, which has a stiffness of approximately 1 MPa [30].

To our knowledge, only one recent study has been conducted in healthy and atherosclerotic human coronary arteries [31]. This report showed almost similar stiffness (average = 10.7 kPa) to the ones we observed (median = 11.0 kPa) for snap-frozen healthy human coronary media nano-indented under liquid conditions at room temperature (electronic supplementary material, §S3), confirming the appropriateness of this experimental approach to interrogate human coronary arteries. With respect to plaque stiffness, our results for lipid-rich areas (median = 2.2 kPa) are comparable to those previously reported [31] (average = 2.7 kPa) in advanced human atherosclerotic plaques. The greatest difference between our results and those published previously, relate to the stiffness of fibrous cap. Previous work reported that fibrous caps were 30% stiffer than healthy coronary media, whereas we found that fibrous caps in our porcine model were 45% less stiff than healthy porcine coronary media. Potential explanations include the immaturity of the fibroatheromas in our model which developed over 18 weeks compared with longstanding established human advanced plaques. In the aforementioned report [31], the advanced human plaques included regional calcifications, which are a hallmark of advanced plaques [32]. The lack of calcification in the plaques we tested is consistent with the plaque age. Moreover, collagen in the early plaques we tested may not be cross-linked to the extent found in longstanding human plaques, which could also account for the values of fibrous cap stiffness observed in this report. Previous work suggests that the plaques leading to acute coronary syndrome frequently are non-stenotic with a plaque burden less than 50%, raising a potential hypothesis that reduced stiffness of the fibrous cap may contribute to the risk of plaque rupture and acute coronary syndrome during plaque development [33–35].

Comparing stiffness results gained from indentation tests with results of bulk tensile or compressive deformations is challenging. First, strain ranges are often small in indention test; while stress–strain testing can reach up to 40–60% strain resulting in significant strain-stiffening. Stiffness results may also be modality-dependent as, in general, indentation tests give lower stiffness results than tensile [36] and unconfined compression testing [23]. Second, arterial tissue stiffness cannot be completely described with a single value (Young's modulus) as it was done herein. It is well accepted that arterial tissue response is strain-dependent, layer-dependent [37] and direction-dependent [38]. However, there is no single gold standard experimental approach to acquiring these data. Tests using a smaller sized indenter enable more localized measurements, therefore, overcoming the tissue-size problem. Obtaining hyperelastic force indentation data requires advanced contact algorithms [39] and sophisticated analytical-experimental approaches [40,41]. Nano-indentation may provide valuable data for multi-scale biomechanical modelling or tuning hyperelastic constitutive models from bulk testing by according to local variations in composition and properties.

### 4.1. Limitations

There are a number of limitations in this work. Despite using gridded coverslips to aid with the co-registration, there is still no complete control over the indentation area, and it is probable that cellular content was indented frequently. Moreover, the thickness of the tissue section may have introduced some variability into the quantification of collagen content, given that 20 µm thick sections were used for histological analysis as opposed to 7–10 µm thick sections that are commonly used [12,42]. It is therefore probable that collagen signal from focal planes across the entire thickness of the section might have influenced the polarized light images. Due to technical difficulties with the staining procedure for only a subset of the 90 indentation locations (31 locations) collagen density data were derived. A linearly polarized illumination source was used, which is a limitation. However, the lack of circularly polarized light is thought not to affect the quantification of collagen fibre density as much as the visualization of collagen orientation [43]. In this work we focused on collagen content only. We also assumed that collagen fibre density is homogeneous across the thickness of the tissue section.

It is known that the collagen network is three-dimensional [44], although in this work we assumed only planar (two-dimensional) variation of the collagen content. We observed a linear association between collagen fibre density and Young's modulus for healthy arteries (figure 3). When the fibrous cap dataset was added to the healthy dataset, the correlation of collagen fibre density to Young's modulus was moderate (Pearson's *ρ* = 0.53, figure 4*c*). Of note, a recent study [45] showed no correlation was found between collagen content of human carotid fibrous caps, quantified by Picrosirius Red, and mechanical properties measured with uniaxial testing. By contrast, the fibrous caps were stronger in the circumferential direction of the native vessel than the axial direction, implying a more prominent role for fibre orientation in affecting local tissue properties. In another study, Chai *et al*. [13] interrogated human advanced carotid plaques with a micro-indentation protocol featuring a spherical probe. Prior to measurement, they visualized areas with different collagen structure (structured/ unstructured) with a collagen-specific fluorescent probe. They were unable to demonstrate a relationship between metrics of collagen structure and tissue stiffness, as areas with structured collagen (median: 31 kPa) were not significantly stiffer than areas with unstructured collagen (median: 33 kPa).

Another major limitation is that only isotropic and linear mechanical behaviour can be extracted from the measurement data. Young's modulus is a concept derived from linear elasticity and may not be suitable to fully capture the dependencies of the mechanical response of arterial tissue upon strain (hyperelasticity), strain rate (viscoelasticity) and direction (anisotropy). In this work we probed the sample with small indentation speeds to ensure viscosity effects were minimized [12]. Also, as mentioned above, indentation measurements may underestimate the real tissue stiffness [36].

Additional limitations of this study include the limited sample size, particularly on a ROI level. The number of ROIs may have been insufficient in order to extract a robust quantitative relation of collagen fibre density and Young's modulus. Nevertheless, the number of gathered force curves that were collected from healthy and diseased arteries is greater than many previous published reports [12,13,46,47]. Due to technical difficulties with the staining procedure for only a subset of the 90 indentation locations (31 locations), collagen density data were derived. In the future, larger studies should be performed using a more systematic histological quantification of arterial constituents: collagen, elastin, lipid and cell content with serial staining.

Finally, it is known the freezing has a small but significant effect on tissue properties. In this study we assumed that the differences between diseased and healthy tissue were larger and that freezing did affect both types of tissue similarly. Further studies are needed to prove these assumptions.

## 5. Conclusion

We investigated whether collagen content affects the compressive axial mechanical properties of porcine coronary arteries with a robust nano-indentation and co-registration protocol. We found that collagen content correlated moderately with Young's modulus and that the mechanical properties of atherosclerotic plaque were related to local histological microstructure. Porcine models of atherosclerosis can provide a platform for refinement of material testing protocols for incorporation into *in vivo* biomechanical models of coronary arteries.

## Data Availability

Data are available from Zenodo. Supplementary material is available online [48].
